# Quantitative T_2_ MRI is predictive of neurodegeneration following organophosphate exposure in a rat model

**DOI:** 10.1038/s41598-020-69991-z

**Published:** 2020-08-03

**Authors:** Kevin Lee, Sara Bohnert, Matthew Bouchard, Cory Vair, Jordan S. Farrell, G. Campbell Teskey, John Mikler, Jeff F. Dunn

**Affiliations:** 1 Hotchkiss Brain Institute, Cumming School of Medicine, University of Calgary, Calgary, AB Canada; 2 Department of Radiology, Cumming School of Medicine, University of Calgary, Calgary, AB Canada; 3 Department of Clinical Neuroscience, Cumming School of Medicine, University of Calgary, Calgary, AB Canada; 4 Department of National Defence, Defence Research and Development Canada- Suffield Research Centre, Alberta, Canada; 5 Department of Neurosurgery, Stanford University, Stanford, CA USA; 6 Faculty of Medicine, Experimental Imaging Centre, Teaching Research and Wellness Building, University of Calgary, 3330 Hospital Drive, Calgary, AB T2N 4N1 Canada

**Keywords:** Cell death in the nervous system, Predictive markers, Magnetic resonance imaging, Epilepsy, Neurotoxicity syndromes

## Abstract

Organophosphorus compounds, such as chemical warfare nerve agents and pesticides, are known to cause neurological damage. This study measured nerve agent-related neuropathology and determined whether quantitative T_2_ MRI could be used as a biomarker of neurodegeneration. Quantitative T_2_ MRI was performed using a 9.4 T MRI on rats prior to and following soman exposure. T_2_ images were taken at least 24 h prior, 1 h and 18–24 h after soman exposure. Rats were pre- and post-treated with HI-6 dimethanesulfonate and atropine methyl nitrate. A multicomponent T_2_ acquisition and analysis was performed. Brains were stained with Fluoro-Jade C to assess neurodegeneration. Rats exposed to soman developed behavioral expression of electrographic seizures. At 18–24 h after soman exposure, significant increases in T_2_, a possible marker of edema, were found in multiple regions. The largest changes were in the piriform cortex (before: 47.7 ± 1.4 ms; 18–24 h: 82.3 ± 13.4 ms). Fluoro-Jade C staining showed significant neurodegeneration 18–24 h post exposure. The piriform cortex had the strongest correlation between the change in relaxation rate and percent neurodegeneration (r = 0.96, p < 0.001). These findings indicate there is regionally specific neurodegeneration 24 h after exposure to soman. The high correlation between T_2_ relaxivity and histopathology supports the use of T_2_ as a marker of injury.

## Introduction

Globally, there are up to two million cases of acute pesticide poisoning annually^1^. Organophosphorus (OPs) compounds are one of the most widely used pesticides and account for 33% of the pesticides used within the United States in 2012^2^. Although new regulations have led to a decrease in OP usage in the United States from 2000^2^, pesticide self-poisoning remains a global health problem in low to middle income countries^1^. The ingestion of OPs may account for over 100,000 deaths per year^1^. More potent forms of OPs include nerve agents that have been developed as chemical warfare agents. Confirmed usage of nerve agents in Syria, in 2013 and 2017, accounted for the death of hundreds of civilians whilst injuring thousands^3^. Nerve agents including tabun, sarin, soman, and VX continue to pose a threat to both civilians and military personnel. Exposure can lead to lacrimation, miosis, tremor, paralysis, convulsive seizures, and respiratory failure, often leading to death if untreated^4^.

A key problem is that following nerve agent exposure, assessing the extent of brain injury is difficult. A non-invasive method that is sensitive to nerve agent related pathology would allow for the monitoring of patients and determining the efficacy of nerve agent treatment protocols. Current treatment protocols are effective in protecting against lethality; however, they may not prevent nerve agent-related neuropathology and associated long-term sequelae. MRI has been used to assess brain pathology post OP exposure^5–12^ but few studies have directly correlated the MR changes to histopathology^7,8,10^. Diffusion MRI provides contrast that relates to cell swelling, changes to myelin, and water content^13^. In models of OP exposure, diffusion MRI has shown contrast that correlates with histological damage detected with hematoxylin and eosin^7,8^ or hemalun-phloxine^10^, and neuroinflammation detected with ionized calcium binding adaptor molecule 1 (IBA1) and glial fibrillary acidic protein (GFAP)^8^. The main contributor to nerve agent-related neurological damage is believed to be from prolonged nerve agent-induced seizure activity^14^. Nerve agents irreversibly inhibit acetylcholinesterase which leads to hyperactivity of cholinergic neurons in the piriform cortex and medial septum^15^. This hyperactivity initiates a cascade that recruits glutamatergic neurons in the temporal lobe causing the onset of seizures^4,16^. Prolonged nerve agent-induced seizures have been shown to cause consistent damage in the cerebral cortex, piriform cortex, amygdala, thalamus, and hippocampus^17^.

Quantitative T_2_ imaging (qT_2_) can be used to study the neuropathology following soman-induced convulsive seizures as it is sensitive to the changes in water content (edema) in the brain microstructure. Previous T_2_ relaxivity studies using soman have mainly focused on using signal intensity^7,9^ or change in T_2_ time^5,6^ to identify regions of damage followed by histology to validate the damage^5–7,9^. However, there have been no studies published that have attempted to correlate T_2_ relaxivity to the percentage of neurodegeneration following soman exposure.

This study used quantitative T_2_ MRI to correlate the severity and regional specificity of neurodegeneration following soman exposure. Soman is typically used to study the neuropathology of nerve agents since it is the fastest aging (form an irreversible bond with acetylcholinesterase) nerve agent^18^ and produces consistent convulsive seizures in defined models. Aging occurs within 2–3 min compared to hours for other nerve agents^18^, which introduces complexity in treating soman exposure. Therefore, treatments that are effective against soman are often effective, if not more so, against other nerve agents^19^.

The first objective of the study was to use qT_2_ MRI to identify structural changes in the brain 1 h and 18–24 h after a convulsive dose of soman. The second objective was to correlate the changes in qT_2_ MRI to the severity of neurodegeneration using histology. We hypothesized that soman-induced convulsive seizures would cause edematous injury and neurodegeneration within 24 h that could be consistently detected using qT_2_ MRI. Establishing the sensitivity of qT_2_ MRI to soman-related neuropathology will be useful as a diagnostic biomarker and facilitate the application to examining the efficacy of treatments in the future.

## Methods

### Experimental design

Previously, we determined that 90 µg/kg (subcutaneous) of soman consistently induced convulsive seizures in a rodent model^20^. HI-6 dimethanesulfonate (HI-6) (125 mg/kg) and atropine methyl nitrate (AMN) (20 mg/kg), were dissolved in saline, and given as pre- and post-treatment (volume of 1 ml/kg; intraperitoneal) to reduce peripheral soman-related symptoms thereby increasing animal throughput. HI-6 reactivates AChE by breaking the phosphate bond between AChE and soman before the aging process^21^. AMN is a competitive inhibitor of ACh at muscarinic receptors, which reduces peripheral cholinergic symptoms^22^. HI-6 and AMN are poor at crossing the blood–brain barrier (BBB), hence have minimal therapeutic benefit for seizure control.

For MRI, rats (n = 23) were anaesthetized with a mixture of 1–2.5% isoflurane, delivered in 70% nitrogen and 30% oxygen. Baseline qT_2_ MRI was performed using a 9.4 T MRI at least 24 h prior to saline or soman treatment. Pre-treatment was administered 20 min prior to soman exposure. For safety during soman exposure, rats were lightly anaesthetized with isoflurane for a maximum of 2–3 min and injected (25G 5/8 needle, volume of 0.4 ml/kg) subcutaneously with soman (90 µg/kg) at the scruff of the neck. The injection site was immediately decontaminated with Reactive Skin Decontamination Lotion and the rat was placed in an observation cage. Rats were observed for the development of soman-related symptoms based on the Suffield rating scale^20^ and behavioral seizures using the Racine scale^23^. An additional three doses of the post-treatment regimen were administered at 20-min intervals (intraperitoneal).

Separate groups of soman treated rats were imaged at 1 h (n = 9) or 18–24 h (n = 10) after exposure. Rats that were given saline were imaged at 1 h and 24 h (n = 4). To account for the neuroprotective properties of isoflurane^24^, the brain tissues of soman treated rats were preserved through fixation (see “Tissue preparation and histology” section) immediately after imaging. The brain tissues of saline treated rats were collected at 24 h. Fluoro-Jade C histochemistry was performed and level of staining quantified as an indicator of neurodegeneration.

### Animals

Animal care protocols were approved by the University of Calgary Animal Care committee and met the Canadian Council of Animal Care (CCAC) guidelines. Rats were closely monitored daily by qualified staff to ensure animal welfare standards were maintained.

Male Sprague–Dawley rats, weighing 240–300 g, were obtained from Charles River Laboratories (Montréal, QC, Canada). Rats were housed in pairs at the University of Calgary Animal Care Facility (Calgary, AB, Canada) in an environmentally controlled room on a 12-h light/dark cycle. Access to food and water ad libitum. All experiments were performed following a minimum acclimatization period of 5 days in their new environment and to human interactions.

### Soman and treatments

(±) Soman (*O*-pinacolyl methylphosphonofluoridate; CAS 96-64-0), HI-6 dimethanesulfonate (CAS 144252-71-1), and Reactive Skin Decontamination Lotion were provided by the Defence Research and Development Canada Suffield Research Centre. Atropine Methyl Nitrate (CAS 52-88-0) was purchased from Sigma Aldrich (Milwaukee, WI, USA).

### Symptom and seizure evaluation

The Suffield Rating Scale was used to evaluate soman-related symptoms^20^ while the Racine scale was used to measure behavioral seizures^23^. Rats treated with soman were monitored and rated every 10 min for up to 2 h. If the rats were rated 4 or higher on the Suffield Rating Scale, additional observations were required until an improvement was observed for at least two consecutive observation periods.

### MRI parameters and analysis

**Figure 1:**
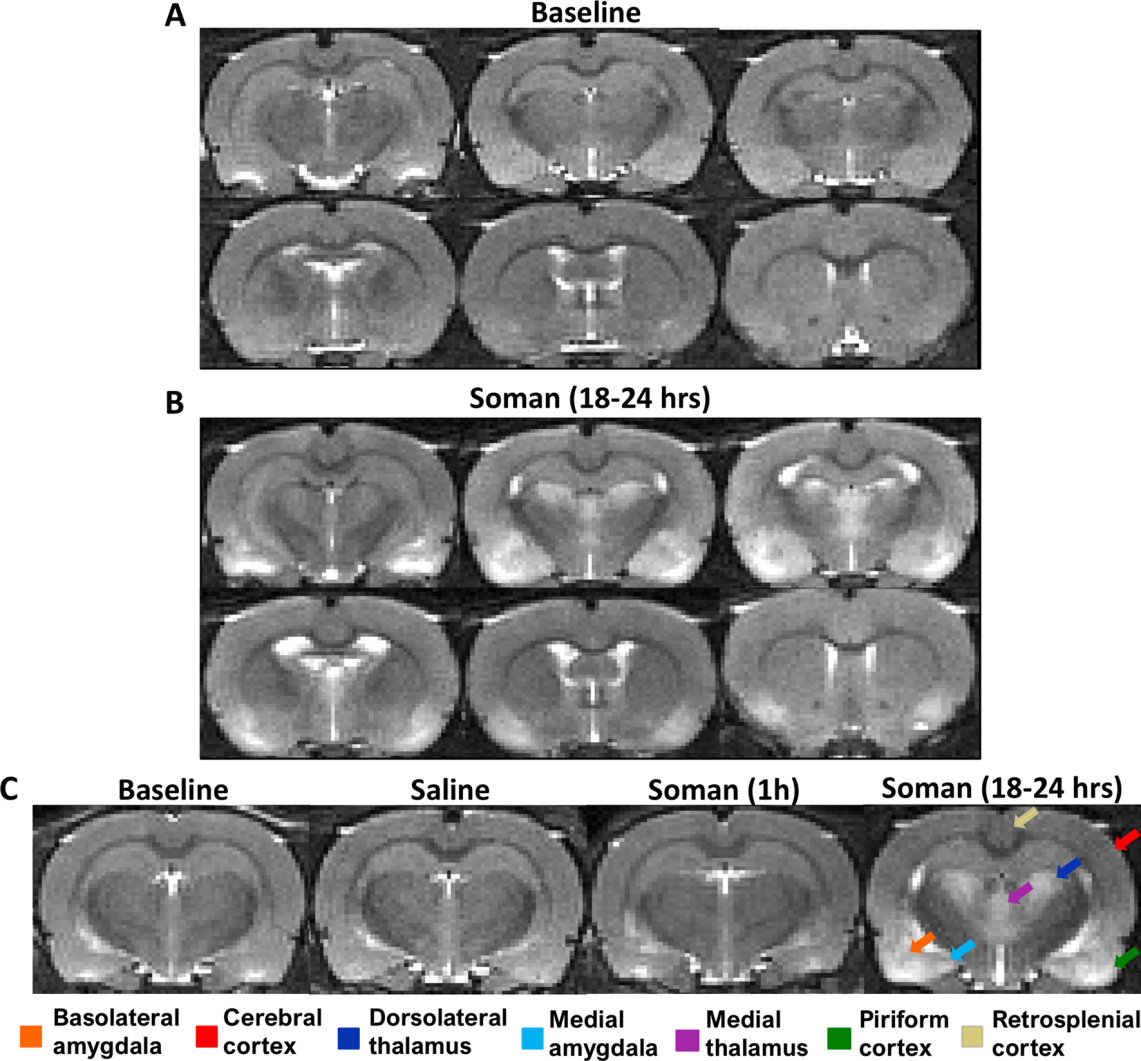
Representative coronal images of the fourth echo (40 ms), (**A**) baseline and (**B**) 18–24 h after soman injection. (**C**) Comparison of baseline, saline, 1 h after soman, and 18–24 h after soman. At 18–24 h after soman exposure, extensive hyperintensive regions were observed in the piriform cortex, cerebral cortex, amygdala, and thalamus (colored arrows). Additionally, ventricular enlargement was seen 18–24 h after soman exposure.

MRI was performed using a 9.4 T Bruker Avance console (Bruker Biospin GmbH, Rheinstetten, Germany) with a 35-mm quadrature volume coil. Rats were anaesthetized with 1–2.5% isoflurane, in 70% nitrogen and 30% oxygen. An in-house built restraining system including ear bars and a bite bar that was used to secure the head and prevent motion artifacts. Scanning protocol involved a multi-slice multi-echo T_2_ sequence: TR = 6,534 ms, 32 echoes were collected with 10 ms echo spacing, 1 average, 20 slices, field of view = 30 × 30 mm^2^, and matrix size = 128 × 128, voxel size = 0.23 × 0.23 × 0.8 mm^3^. Respective baseline images were obtained for each rat followed by either saline or soman injection (Fig. 1). Throughout the image acquisition, temperature and respiration rate were monitored and maintained at normal physiological range (approximately 36.5 °C and 60 breaths per minute).

Multiexponential T_2_ analysis was performed using AnalyzeNNLS (https://sourceforge.net/projects/analyzennls/), which is a compilation of open source MATLAB scripts^25^. The T_2_ relaxation curve was mapped starting with the second echo due to complications with the stimulated echoes. Tissue heterogeneity produces a decay curve composed of multiexponential components. The different components in a multiexponential decay curve can be analyzed by Eq. (1)^26^:

1 $${y}_{i}= \sum_{j=1}^{M}{s}_{j}{e}^{({-t}_{i}/{T}_{2j})}i=\mathrm{1,2},\dots ,N$$

where t_*i*_ is the echo time, M = 120 which are the logarithmically spaced T_2_ times between 5 to 640 ms, N = 32 which represents the total number of echoes, and s_*j*_ represents the relative signal amplitude at the partitioned T_2_ time (T_2j_). The analysis utilizes a non-negative least square (NNLS) algorithm to minimize misfit and smoothing constraint for the T_2_ distribution. This provides a more consistent fit when noise is present^26,27^. We defined the area under the amplitude of the T_2_ curve as three different water compartments depending on the T_2_ values. We used the range of T_2_ < 25 ms for myelin associated water, T_2_ of 25–200 ms for intra/extracellular water, and T_2_ > 2000 ms for cerebrospinal fluid.

**Figure 2:**
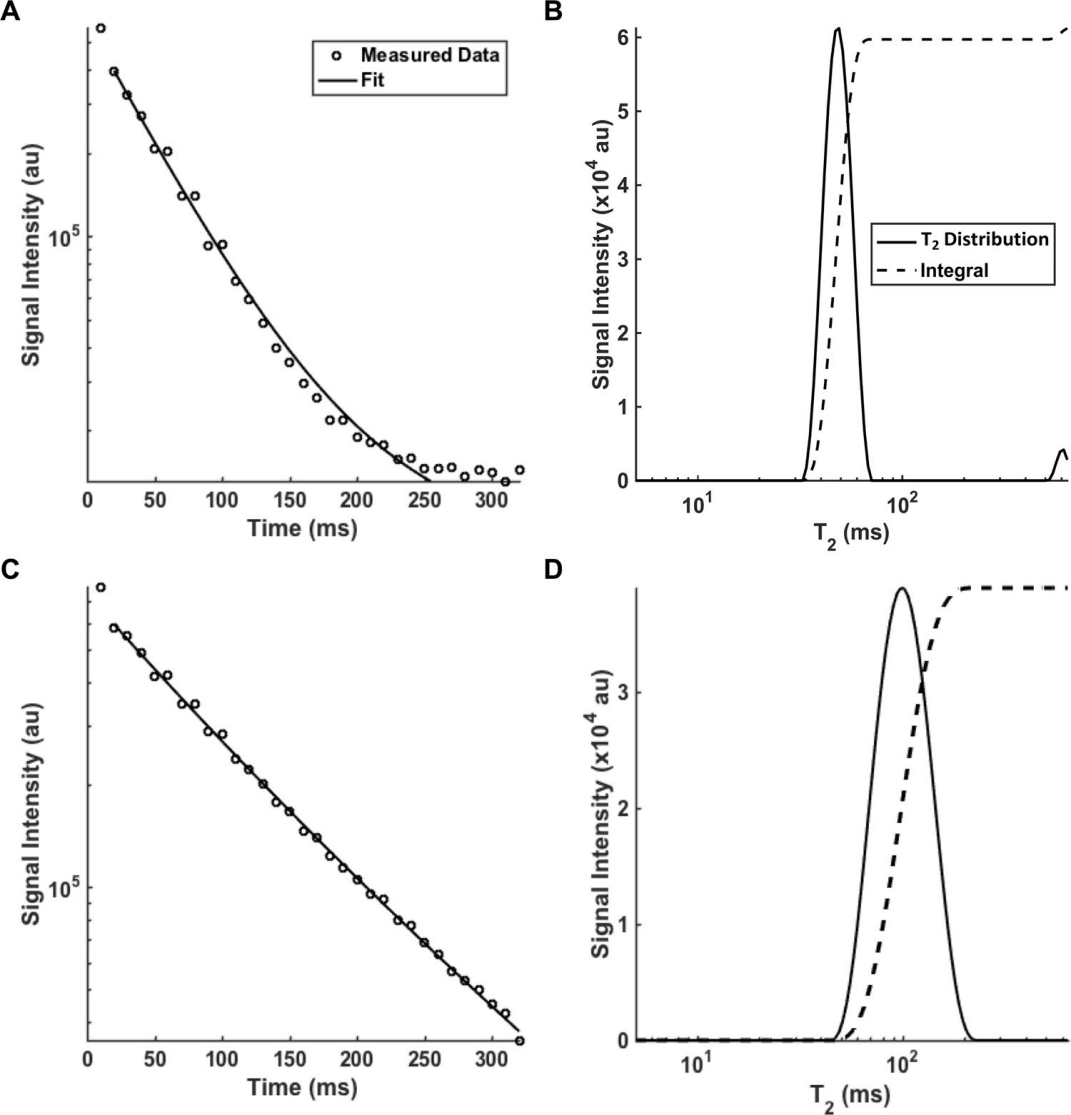
Representative T_2_ fitting and distribution before (**A**,**B**) and 18–24 h after a convulsive dose of soman (**C**,**D**) in the piriform cortex. A, signal intensity decayed by 250 ms. B, the mean geometric T_2_ distribution was 48 ms. (**C**) 18–24 h after soman higher signal intensity was detected with the signal decay by 320 ms. (**D**) 18–24 h after soman the mean T_2_ distribution was 93 ms which widened and shifted to the right.

A rat brain atlas^28^ was used as a reference to manually draw the corresponding regions of interest in the qT_2_ images. Signal changes before and after exposure were measured in the piriform cortex, basolateral amygdala, medial amygdala, medial thalamus, dorsolateral thalamus, cerebral cortex, and retrosplenial cortex. Representative T_2_ fitting and distribution before and 18–24 h after soman-induced seizures in the piriform cortex can be seen in Fig. 2.

### Tissue preparation and histology

Rats were deeply anaesthetized with sodium pentobarbital (200 mg/kg; intraperitoneal) immediately following MRI. To preserve the brain tissue, rats were intracardially perfused with 150 ml of cold 1% phosphate-buffered saline (PBS) and fixed with 200 ml of 4% paraformaldehyde (PFA) solution. Brains were extracted and stored in 4% PFA for 24 h at 4 °C then transferred to a 30% sucrose solution for long-term storage. The brains were sliced to 25 μm thickness using a cryostat (Leica, Biosystems). Slices were placed on electrostatically charged slides and stored at − 80 °C.

To determine the number of neurons in control tissue, saline treated rats were stained for Neuronal Nuclei (NeuN) (no. ab177487, Abcam Inc, ON, Canada). The slides were removed from the freezer and air dried for 30 min. The slides were then incubated for 1-h in a blocking serum composed of 10% horse serum, 1% bovine serum albumin (BSA), 0.1% cold fish skin gelatin (CFSG), 0.5% Triton X-100, and 0.05% Tween-20 in 1 × PBS. Following incubation slides were rinsed in a 0.05% Tween-20 solution in 1 × PBS for 10 min. The primary antibody NeuN, diluted with 5% horse serum, 1% BSA, 0.1% CFSG, and 0.5% TritonX-100 in 1 × PBS, was applied on the slides for 12-h. Slides were once again rinsed in 0.05% Tween-20 in 1 × PBS for 10 min. The secondary antibody Alexa Fluor 594 (no. 711-585-152, Jackson ImmunoResearch Laboratories Inc, PA, USA) was diluted in 1% BSA, 0.1% CFSG, and 1% TritonX-100 solution in 1 × PBS. Slides were incubated with the secondary antibody for 1-h and rinsed in 0.05% Tween-20 in 1 × PBS for 10 min. Throughout the secondary incubation and all subsequent steps, the slides were kept in a dark environment at room temperature. Slides were then fitted with coverslips using Immuno-Mount (Thermo Scientific, ON, Canada).

Tissues were stained using Fluoro-Jade C (no. AG325, Millipore, ON, Canada), which has been shown to selectively stain for degenerating neurons^29^. Frozen sections were rehydrated followed by incubation in 0.06% KMnO_4_ for 20 min. The sections were then drained and rinsed for 2 min with distilled water followed by incubation in 0.00001% Fluoro-Jade C (FJC) solution for 20 min. Following incubation slides were rinsed three times in distilled water and dried with a hair dryer for 5 min in a dark environment. Dried slides were placed in xylene for 5 min, and cover slipped with dibutylphthalate polystyrene xylene (DPX) mounting medium (Sigma Aldrich, Milwaukee, WI, USA).

### Quantification of NeuN and FJC

A rat brain atlas^28^ was used to determine the counting frame in FJC and NeuN stained slices. The stained sections were visualized using the Olympus BX61-DSU microscope. FJC was viewed with a fluorescein isothiocyanate filter at 450–490 nm and NeuN was viewed with a tetramethylrhodamine filter at 488–532 nm. The images were taken using a high-resolution digital camera from MBF Bioscience (Williston, VT, USA). Quantification of FJC and NeuN positive cells was performed manually using Stereo Investigator (version 11, MBF Bioscience). Regions of interests were identified at × 10 magnification (Numerical aperture = 0.24). The counting frame was 400 × 400 μm^2^ in the regions of interest. The FJC and NeuN positive cells per area were calculated via number of cells over the area of the counting frame.

To quantify and compare the extent of neurodegeneration, the heterogeneity in cells per area between regions must be accounted for. As such, we calculated the percentage of neurodegeneration by comparing the FJC and NeuN positive cells per area.

### Statistics

All statistical analyses were performed using R 3.5.0^30^ or MATLAB 2018a Statistics and Machine Learning Toolbox (Mathworks, Natick, MA, US). There were three groups (saline, 1 h after soman, and 18–24 h after soman), each imaged twice before and after saline or soman exposure. A general linear model was performed evaluating the changes in T_2_ after exposure. Group (saline, soman 1 h, soman 18–24 h), treatment (before, after exposure), and brain regions were entered as fixed effects. This was followed by Tukey’s honestly significant difference post-hoc test. For the linear regression, the relaxation rate (R_2_) was calculated using $R_{2}=1/T_{2}$. The relationship between changes in R_2_ vs. percentage of neurodegeneration was determined through linear regression to calculate the correlation coefficient (r), and equation of the line. We excluded one rat from the linear regression because tissues did not adhere to the slides and FJC staining could not be assessed. Statistical significance was determined a priori using α = 0.05.

## Results

Consistent with our previous study^20^, rats (n = 19) exposed to soman developed generalized tonic–clonic seizures within 7–21 min post exposure. These rats continued to exhibit intermittent mild tremors with rhythmic head movement until imaging at 1 h (n = 9) or 18–24 h (n = 10). As expected, rats (n = 4) treated with saline did not exhibit behavioral abnormalities.

To consider qT_2_ MRI as an accurate depicter of brain damage following soman-induced seizures, the images that it produces need to clearly depict the regions of damage. Compared to the baseline (Fig. 1A), at 18–24 h after soman exposure we found clearly defined hyperintensive regions in the basolateral amygdala, medial amygdala, dorsolateral thalamus, medial thalamus, and piriform cortex (Fig. 1B). Whereas more subtle hyperintensity was observed in the cerebral cortex and retrosplenial cortex (Fig. 1B). The cohort 18–24 h after soman had notable changes that were easily identifiable, while there were no observable changes in saline treated and 1 h after soman exposure groups (Fig. 1C). As there were substantive changes found 18–24 h after soman exposure, the change in T_2_ relaxation time was quantified in the regions of interest, using a multiexponential T_2_ decay analysis.

Multiexponential T_2_ analysis was used to obtain the T_2_ distribution and decay curves because it provides enhanced pathological specificity compared to a monoexponential T_2_ analysis^31^. A representative analysis using the piriform cortex as an example is shown in Fig. 2. As expected, at 18–24 h after soman exposure the T_2_ distribution was higher and wider with a rightward shift (Fig. 2B,D). The baseline had a geometric mean T_2_ of 48 ms (Fig. 2B) while 18–24 h after soman exposure the T_2_ was 93 ms (Fig. 2D). We did not see any peaks between 200 and 2000 ms, which is consistent with previous observations^32–34^.

**Figure 3:**
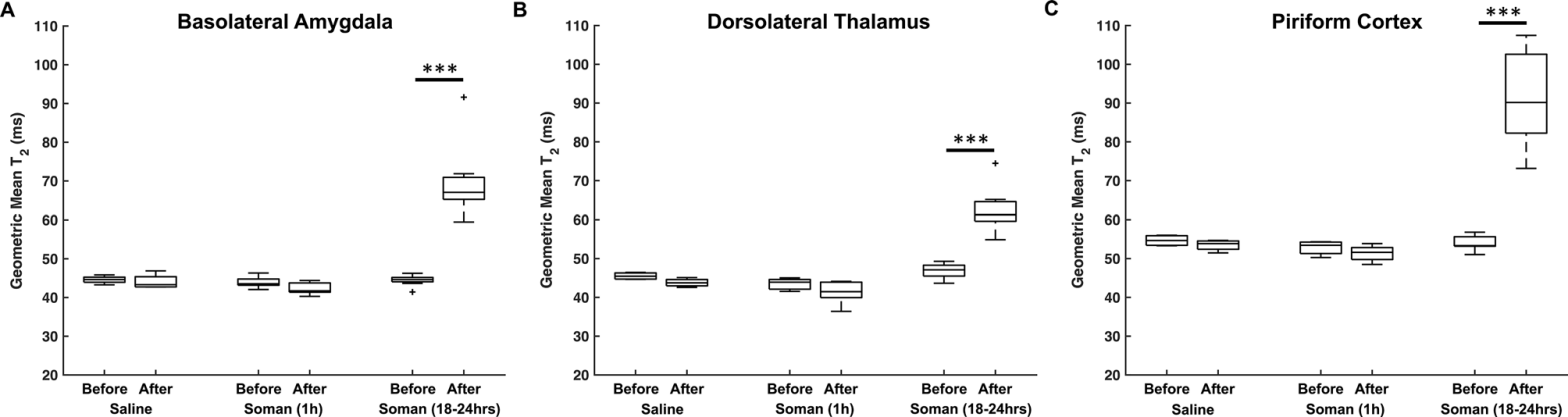
The effects of soman exposure on T_2_ relaxation time before exposure, 1 h after soman (n = 9), or 18–24 h after soman (n = 10). (**A**–**C**) there was no significant difference 1 h after soman exposure from the respective baseline. There was a significant difference 18–24 h after soman exposure from the respective control in the basolateral amygdala (t = 14.8, df = 280, p < 0.001), dorsolateral thalamus (t = 12.5, df = 280, p < 0.001), and piriform cortex (t = 20.5, df = 280, p < 0.001). There was no significant difference in the saline treated group (n = 4). Rats were imaged at least 24 h before treatment. Black bars are the maximum and minimum T_2_ time. Middle black line is the median and the plus signs are outliers. The baseline for each group served as their own respective control. *p < 0.05, **p < 0.01, ***p < 0.001.

We quantified the changes in T_2_ relaxation time after soman-induced seizures to determine the damage in the regions of interest (Table 1). There was a three-way interaction between group, treatment, and brain region (F_12, 280_ = 11.71, p < 0.001). Post-hoc test was performed and revealed that there was no significant change pre and post saline exposure (n = 4) in every brain region (Fig. 3, Supplementary Fig. S1). Additionally, at 1 h after soman exposure (n = 9) there was no significant change before and after soman in regions of interest (Fig. 3, Supplementary Fig. S1). At 18–24 h after soman exposure (n = 10), there was a significant increase in T_2_ in the basolateral amygdala by 36% (t = 14.8, df = 280, p < 0.001), dorsolateral thalamus by 35% (t = 12.5, df = 280, p < 0.001), and piriform cortex by 42% (t = 20.5, df = 280, p < 0.001) (Fig. 3). A significant increase in T_2_ was also found in the cerebral cortex by 20% (t = 5.8, df = 280, p < 0.001), medial amygdala by 21% (t = 7.3, df = 280, p < 0.001), and medial thalamus by 29% (t = 10.2, df = 280, p < 0.001) (Supplementary Fig. S1). Quantification of T_2_ reveals that the piriform cortex had the most severe damage followed by the basolateral amygdala and dorsolateral thalamus.

**Table 1:** Mean T_2_ times and neurodegeneration in different anatomical brain structures in rats before and after soman exposure were calculated.

	Saline (n = 4)			Soman—1 h (n = 9)			Soman—18–24 h			Degenerating neurons (%)
	T_2_ (ms)		FJC + cells/area (#cells/mm^2^)	T_2_ (ms)		FJC + cells/area (#cells/mm^2^)	T_2_ (ms)		FJC + cells/area (#cells/mm^2^)	
	Before	After		Before	After		Before	After		
BasolateraI amygdala	44.6 ± 1.1	44.1 ± 2.0	0	43.9 ± 1.3	42.3 ± 1.5	0	44.5 ± 1.3	69.4 ± 8.6***	519 ± 215	55
Cerebral cortex	39.3 ± 0.3	38.0 ± 0.7	0	39.7 ± 1.4	37.6 ± 1.3	0	38.7 ± 1.5	48.4 ± 7.1***	381 ± 165	23
Dorsolateral thalamus	39.8 ± 0.7	38.1 ± 0.8	0	37.3 ± 1.8	36.9 ± 2.1	0	38.4 ± 1.3	59.4 ± 6.2***	521 ± 61	69
Medal amygdala	46.4 ± 1.0	46.4 ± 1.8	0	45.4 ± 1.4	46.9 ± 2.2	0	46.1 ± 1.0	58.4 ± 3.2***	747 ± 205	50
Medial thalamus	42.2 ± 0.6	42.8 ± 1.0	0	42.8 ± 1.0	42.9 ± 1.7	0	42.8 ± 1.4	59.9 ± 6.0***	669 ± 181	60
Piriform cortex	48.2 ± 0.4	47.6 ± 0.5	0	46.8 ± 1.9	46.9 ± 1.3	0	47.7 ± 1.4	82.3 ± 134***	701 ± 139	76
Retrosplenial cortex	39.4 ± 0.9	37.2 ± 1.5	0	36.4 ± 2.0	36.9 ± 1.2	0	37.3 ± 1.3	39.1 ± 1.6	291 ± 110	25

Percent degenerating neurons were calculated by comparing the FJC + cells/area density in soman treated rats and NeuN + cells/area in saline treated rats.

Data are mean ± standard deviation.

General linear model. Statistical significance were calculated using each rats respective baseline images prior to treatment.

Significance: *p < 0.05, **p < 0.01, ***p < 0.001.

**Figure 4:**
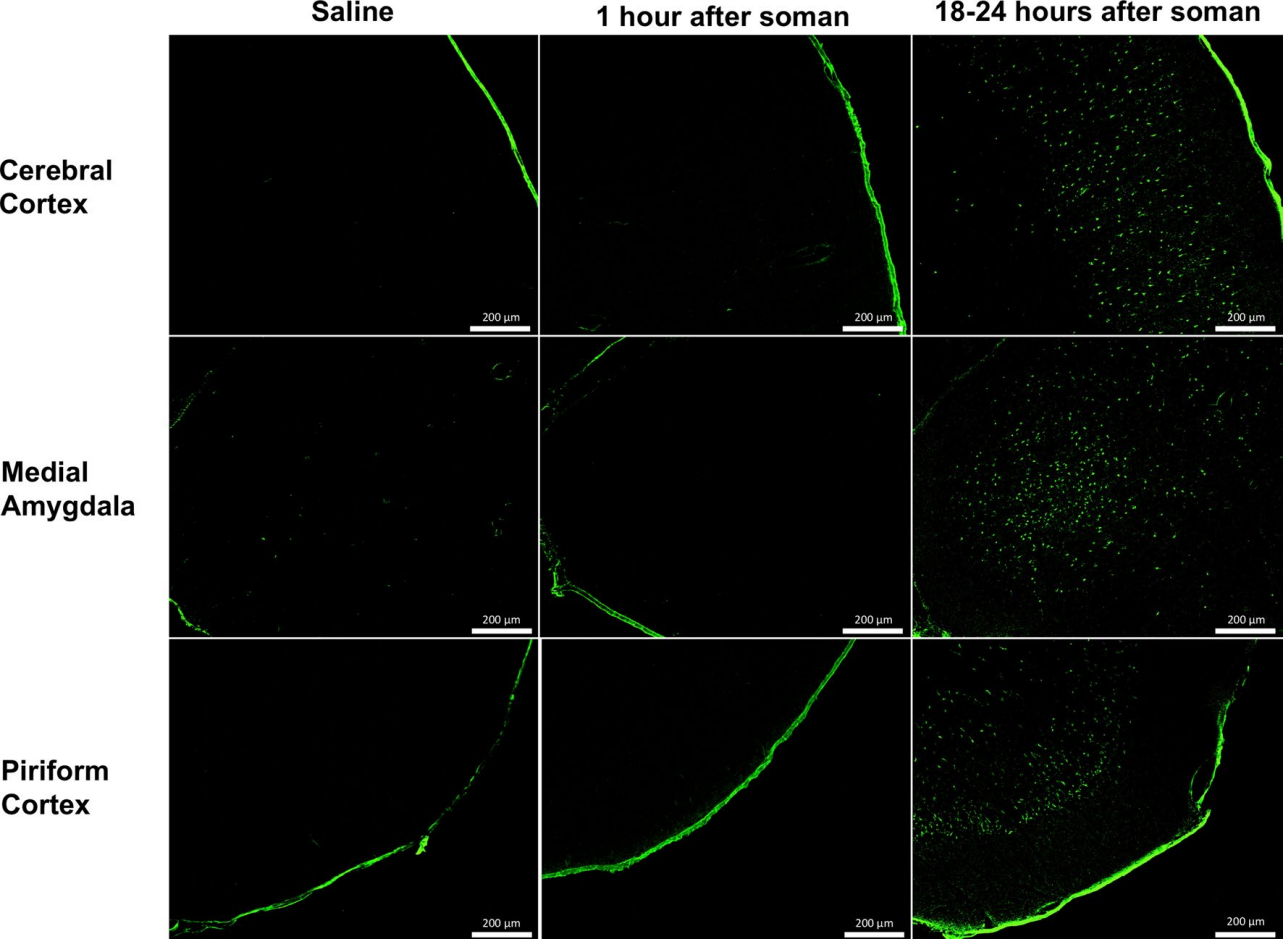
Effect of soman-induced seizures on neuronal cells following 18–24 h after soman exposure in the cerebral cortex, medial amygdala, and piriform cortex. Rats were perfused and fixed immediately after MR imaging and stained using Fluoro-Jade C, which is a marker for neurodegeneration. Fluoro-Jade C positive cells were manually quantified in regions corresponding to the T2-weighted MR images.

Using FJC, which is a stain for neurodegeneration^29^, we validated the damage found in the regions of interest when using qT_2_ MRI. The qualitative assessment suggests that there was no neurodegeneration in saline treated rats or in rats examined 1 h after soman exposure (Fig. 4, Supplementary Fig. S2). While at 18–24 h after soman exposure, there were a high number of FJC positive cells in the regions of interest (Fig. 4, Supplementary Fig. S2). To quantify this observation, we analyzed the FJC positive cells per area in the regions of interest (Table 1). We found the medial amygdala had the highest neurodegeneration per area of 747 ± 205 cells per mm^2^ (mean ± SD) while the retrosplenial cortex had the lowest neurodegeneration per area of 291 ± 110 cells per mm^2^. However, when the heterogeneity in cells per area between regions were accounted for, using NeuN, we found the piriform cortex had the highest percentage of neurodegeneration of 76% while the cerebral cortex had the lowest percentage of 23%. These finding suggests there may be heterogeneity in the progression of neurodegeneration that are region dependent and are not reflective of the T_2_ relaxation time.

**Figure 5:**
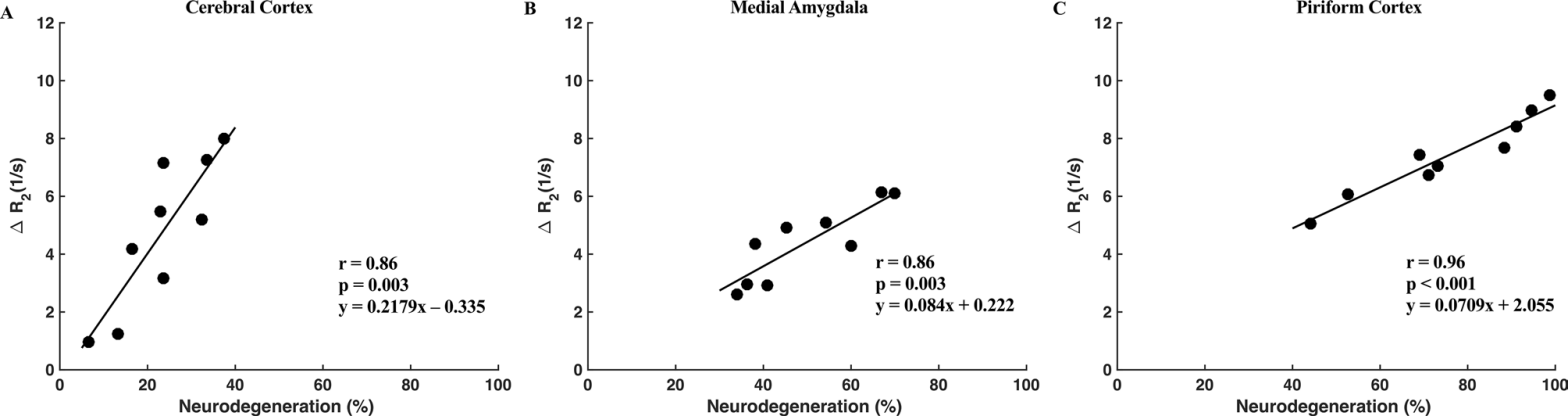
Effect of soman-induced seizures on the rate of relaxation (R_2_) correlated to the percentage of neurodegeneration in the cerebral cortex, medial amygdala, and piriform cortex. (**A**–**C**) significant correlation was found in the cerebral cortex (r = 0.86, p = 0.003), medial thalamus (r = 0.86, p = 0.003), and piriform cortex (r = 0.96, p < 0.001). Each point represents one rat.

To determine the efficacy of qT_2_ MRI in delineating soman-related brain damage, the changes in the relaxation rate (R_2_ = 1/T_2_) were correlated to the percentage of neurodegeneration in regions of interest. The percentage of neurodegeneration was used to account for heterogeneity between regions. We correlated with R_2_ as the relaxation rate changes linearly with factors that influence relaxation of water^35^. Since there were no FJC positive cells in saline treated or 1 h after soman exposure, only the 18–24 h after soman exposure group (n = 9) was analyzed. There was a significant correlation in the cerebral cortex (r = 0.86, p = 0.003 with a slope of 0.013), medial amygdala (r = 0.86, p = 0.003 with a slope of 0.006), and piriform cortex (r = 0.96, p < 0.001 with a slope of 0.008) (Fig. 5). Additional correlations are shown in the supplementary information: dorsolateral thalamus (r = 0.76, p = 0.02, with a slope of 0.014), and medial thalamus (r = 0.78, p = 0.014 with a slope of 0.0065) (Supplementary Fig. S3). Notably, there was no significant correlation in the basolateral amygdala (r = 0.07, p = 0.87 with a slope of 4.99 × 10^–4^), and retrosplenial cortex (r = 0.63, p = 0.07 with a slope of 0.004). The piriform cortex had the highest correlation coefficient of 0.96 followed by the cerebral cortex and medial amygdala with a correlation coefficient of 0.86.

A potential challenge in predictive MRI is the inter-subject variability. The heterogeneity of T_2_ relaxation times in the baseline was within 2 ms in each brain region, which is a coefficient of variation of less than 6%. At 1 h after soman exposure, when no neurodegeneration was observed, the standard deviation was 2.2 ms, which is a coefficient of variation less than 5%. When high levels of neurodegeneration were noted, at 18–24 h after soman, the standard deviation was 13.4 ms, with a coefficient of variation of 16%.

## Discussion

T_2_ has been widely used in MRI to detect major changes in water content and tissue pathophysiology in preclinical models such as cancer^36,37^ and stroke^38,39^. Much of the previous T_2_ related literature quantifies T_2_ using less than seven echo times to describe a relaxation curve following soman exposure^5,6,12^. In order to quantify the T_2_ more accurately, more echoes were utilized to fit the decay curve. The brain is a heterogeneous structure mainly composed of water, lipids, and proteins. The decay curve can be processed as a multiexponential function to describe different T_2_ components representative of the microstructures in the brain. The complex nature of the different water environments results in multiple T_2_ decay times within a single voxel. In utilizing a NNLS T_2_ fitting algorithm, the T_2_ decay curve can be separated into the respective water compartments that contribute to the total T_2_ signal. Analysis using NNLS algorithm is advantageous as it makes no assumptions a priori on the number of water compartments that make up the T_2_ decay curve^26^. Therefore, rather than assuming a set number of water compartments through a few number of exponentials, NNLS can analyze relaxation data in a continuous model^26^. Compared to a discrete spectrum a continuous spectrum considers slow and fast decaying protons in addition to intermediate decaying protons, which may arise from an interaction between compartments^40^, thereby better modelling biological samples. In a healthy brain with high signal to noise, three components are commonly seen, a short component associated with myelin water, a medium component associated with intra/extracellular water, and a long component associated with CSF^31,32,34^. Although a healthy brain can be accurately modelled with three components, in pathological states, having a large number of relaxation components can provide a more accurate overview on the changes in microstructures. For a discussion on the pros and cons of using NNLS see paper by Whittall and MacKay^26^.

In our study, we rarely saw a short T_2_ component in the grey matter. This component has been attributed to water interacting with myelin or myelin water fraction^34^. It is common in animal studies to only detect one component in the grey matter. This is because of the low myelin content^32^. This study focused on detecting cerebral edema as well as the pathophysiology of tissue damage in the grey matter following soman exposure.

At 18–24 h post-soman exposure, rats had cerebral edema and neurodegeneration in the amygdala, cerebral cortex, piriform cortex, and thalamus based on increases in T_2_. These findings correspond with previous studies where cerebral edema was observed 24 h after OP-induced seizures^5–7,10–12,17^ and is thought to be a combination of both cytotoxic and vasogenic edema^11^. The edema following soman-induced seizures likely developed from the damage sustained through excitotoxicity^41–43^, oxidative stress^44,45^ and neuroinflammation^46–48^. When the neurons are unable to recover from the damage, they undergo degeneration. This corresponds to our study where the edematous regions showed extensive neurodegeneration using FJC (Fig. 3, Supplementary Fig. S2). FJC staining was used to assess neurodegeneration as this stain shows specificity towards degenerating neurons^29^. Normalization using NeuN has shown the piriform cortex to have the highest level of neurodegeneration, which is similar to what was observed using qT_2_. Therefore, we speculate that the extent of neurodegeneration may mediate cerebral edema.

T_2_ is often used as a marker of edema but it also shows changes in cell pathophysiology. The strong correlation with FJC indicates that either edema strongly correlates with neurodegeneration or that T_2_ is detecting changes from two processes, cell integrity and edema. Regardless, qT_2_ is proving to be a sensitive metric of neurodegeneration associated with edema. Based on a study using sarin, changes in the BBB occurs as early as 2 h but leakage does not occur until 24 h^49^. The severity of localized BBB disruption may be proportional to the inflammatory cytokines released from degenerating neurons following soman-induced seizures. Cytokine release including interleukin (IL)-6, IL-1 α, IL-1 β, and tumor necrosis factor- α following soman-induced seizures^48,50^ were found to increase the permeability of the BBB^51^. The increased permeability may allow neutrophil infiltration, which can further exacerbate the initial injury and cause BBB leakage. The piriform cortex was shown to have a high number of neutrophil infiltrations at 24 h following soman-induced seizures^47^ which matches the time point where vasogenic edema is expected to occur. A study using a nerve agent substitute, diisopropyl fluorophosphate, found a correlation between diffusion MRI (apparent diffusion coefficient) and neuronal necrosis and neuroinflammation^8^. Thus, the vasogenic edema that we observed at 18–24 h may be mediated through neuroinflammation. As a result, we were able to demonstrate that qT_2_ was able to detect the severity of neurodegeneration non-invasively.

The high degree of correlation between R_2_ and neurodegeneration following soman exposure demonstrates the feasibility of qT_2_ MRI as a marker of injury (Fig. 5, Supplementary Fig. S3). The cerebral cortex, medial amygdala, and piriform cortex showed consistent pathology with T_2_ (Fig. 3, Supplementary Fig. S1) and histology (Fig. 4, Supplementary Fig. S2). However, there appears to be significant regional specificity of neurological damage following soman-induced seizures. In particular the piriform cortex had the highest correlation between R_2_ and percent neurodegeneration among the regions of interest (Fig. 5c). The difference in the level of predictability may be due to the regional susceptibility to seizures or the time course for the development of neuropathology. The piriform cortex was speculated as the site of seizure onset following OP exposure^15^ which may allow more time for edema to develop. This region has been shown to also play an important role in initiating and mediating seizure activity in rodents^52^. Based on qT_2_, regional differences in the pathophysiology post soman exposure, and changes in T_2_ that are proportional to the magnitude of neurodegeneration can be clearly detected. This method of imaging can be useful for diagnosis of nerve agent mediated brain injury, assessment of efficacy of novel treatments, and provide a critical time course of recovery.

One might expect that the early time course of T_2_ could be predictive of long-term pathology. Prior to this study, the earliest time point following soman-induced seizures where T_2_ was measured was 3 h^12,13^ and 6 h^32^. No changes were reported^5,6,12^. We also found no significant reduction in T_2_ 1 h after soman, although there was a trend in the cerebral cortex and basolateral amygdala. Interestingly, a study in a febrile seizure model found a decrease in T_2_ in the basolateral amygdala two hours post seizures^53^. A decrease in T_2_ in the basolateral amygdala was predictive of epileptogenesis^53^. Spontaneous recurrent seizure have also been detected following soman exposure^54^.

The decrease in T_2_ found in febrile seizure model may be due to hypoxia, which results in an increase in deoxyhemoglobin^55^. We have previously found a decrease in cerebral blood flow throughout the brain 1-h after soman exposure^56^. Despite such a decrease in cerebral blood flow either from neurovascular uncoupling^57^ or transient vasoconstriction^20,58^, T_2_ did not change. The growing evidence of physiological changes at this time point suggests that early changes in T_2_ may be difficult to detect.

The present study demonstrates the value of qT_2_ MRI as a marker for neurodegeneration following OP exposure. The extent of neurodegeneration from seizures are often difficult to determine as the pathologies are transient. MRI non-invasive characteristics has the advantage that it could be undertaken over a time course which would help describe regional progression of injury. We suggest that qT_2_ MRI, as a non-invasive assessment of acute OP toxicity, has the potential to be a paradigm for determining the effectiveness of treatments aimed at reducing OP related neuropathology. Previous studies have found the neurodegeneration to be the highest at 24 h following OP exposure^17^. Therefore, reducing damage within that time frame may be critical in the prevention of long-term pathologies including the development of spontaneous recurrent seizures^54,59^.

Another area of interest may lie in the translational value of the results as a diagnostic marker. The similar symptomology of nerve agents with both cyanide poisoning and opioid overdose makes diagnosis difficult, especially when time is a critical factor^60^. In our results, we found the high level of changes in the piriform cortex. A closely related structure to the piriform cortex is the insular cortex in human. Victims of the 1995 Tokyo subway sarin attack had decreased brain volume in the insular and temporal cortex, 5 to 6 years following the attack^61^. A recent report on the Havana Syndrome, which suspects OP insecticide exposure, has found BBB leakage in the insular cortex^62^. Both of these studies were conducted using MRI and detected subtle changes. Therefore, we propose that detecting localized damage in the insular cortex using qT_2_ MRI may help clinicians to identify acute OP toxicity.

In the future, qT_2_ MRI should be used to acquire additional time points to elucidate the development of OP related neuropathology over time including both short- and long-term changes. Additionally, the development of neurodegeneration should be compared with other physiological measures including, seizure duration, serum acetylcholinesterase, cerebral blood flow, tissue oxygenation, and BBB leakage.

Using qT_2_ MRI, we demonstrated that T_2_ relaxation time was correlated to neurodegeneration following soman exposure. At 18–24 h after soman, we found that an increase in T_2_, which is indicative of cerebral edema, correlated with neurodegeneration. Although correlations were seen in many different regions, we found the highest correlation in the cerebral cortex, medial amygdala, and piriform cortex. The high degree of correlation and consistency of T_2_ relaxation time provide evidence that qT_2_ could be used as a biomarker for civilian and military personnel for nerve agent exposure.

## Supplementary information

Supplementary Information

## Data Availability

The datasets generated during and/or analysed during the current study are available from the corresponding author on reasonable request.
