# Supplementary material for: Quantitative T2 MRI is predictive of neurodegeneration following organophosphate exposure in a rat model
Source: Sci Rep. 2020 Aug 3;10:13007. doi: 10.1038/s41598-020-69991-z (PMC7400670; doi:10.1038/s41598-020-69991-z)
Supplement: Supplementary file 1 — Supplementary Information [file 41598_2020_69991_MOESM1_ESM.docx]

Quantitative T_2_ MRI is predictive of neurodegeneration following organophosphate exposure in a rat model

Kevin Lee^1, 2, 3^, Sara Bohnert^4^, Matthew Bouchard^1, 2, 3^, Cory Vair^4^, Jordan S. Farrell^1, 5^, G. Campbell Teskey^1^, John Mikler^4^, and Jeff F. Dunn*^1, 2, 3^

1: Hotchkiss Brain Institute, Cumming School of Medicine, University of Calgary, Calgary, Alberta, Canada

2: Department of Radiology, Cumming School of Medicine, University of Calgary, Calgary, Alberta, Canada

3: Department of Clinical Neuroscience, Cumming School of Medicine, University of Calgary, Calgary, Alberta, Canada

4: Defence Research and Development Canada- Suffield Research Centre, Department of National Defence, Alberta, Canada

5: Department of Neurosurgery, Stanford University, Stanford, California, USA

Author to whom correspondence should be addressed

Email: [dunnj@ucalgary.ca](mailto:dunnj@ucalgary.ca)

Experimental Imaging Centre, Teaching Research and Wellness Building, University of Calgary, Faculty of Medicine, 3330 Hospital Drive, Calgary, Alberta, Canada, T2N 4N1


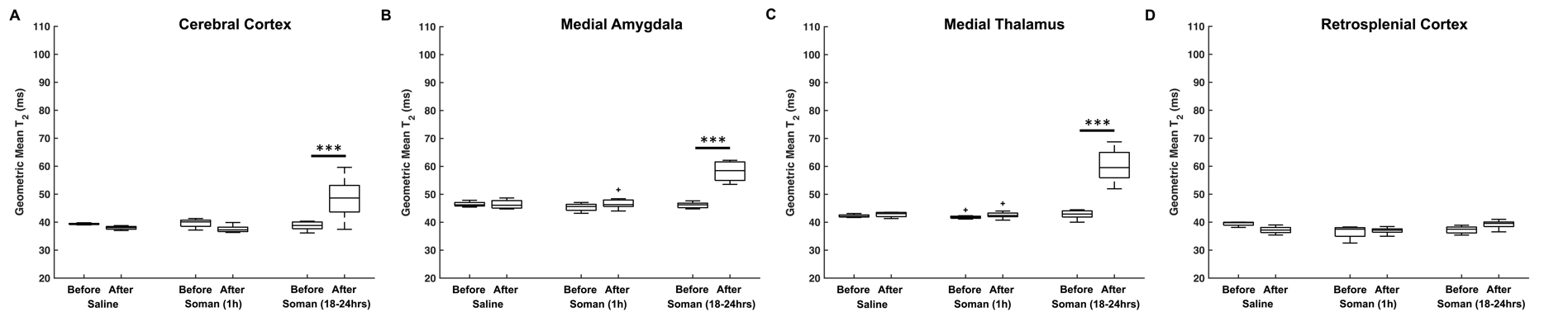


**Supplementary Figure S1.** The effects of soman exposure on T_2_ relaxation time in the cerebral cortex, medial amygdala, medial thalamus, and retrosplenial cortex before, 1 hour after soman (n = 9), and 18-24 hours after soman (n = 10). A-D, there was a no significant change 1 hour after soman exposure compared to their respective pre exposure. At 18-24 hours after soman exposure, there was a significant increase in the T_2_ relaxation time in the cerebral cortex (t = 5.8, df = 280, p < 0.001), medial amygdala (t = 7.3, df = 280, p < 0.001), medial thalamus (p < 0.001, t = 10.22, df = 9), and retrosplenial cortex (t = 10.2, df = 280, p < 0.001). No significant difference in the saline treated group (n = 4). Pre exposure images were acquired at least 24 hours before treatment. The black bars are the maximum and minimum T_2_. Middle black line is the median and the plus signs are outliers. Each group has their respective control. *p < 0.05, **p < 0.01, ***p < 0.001


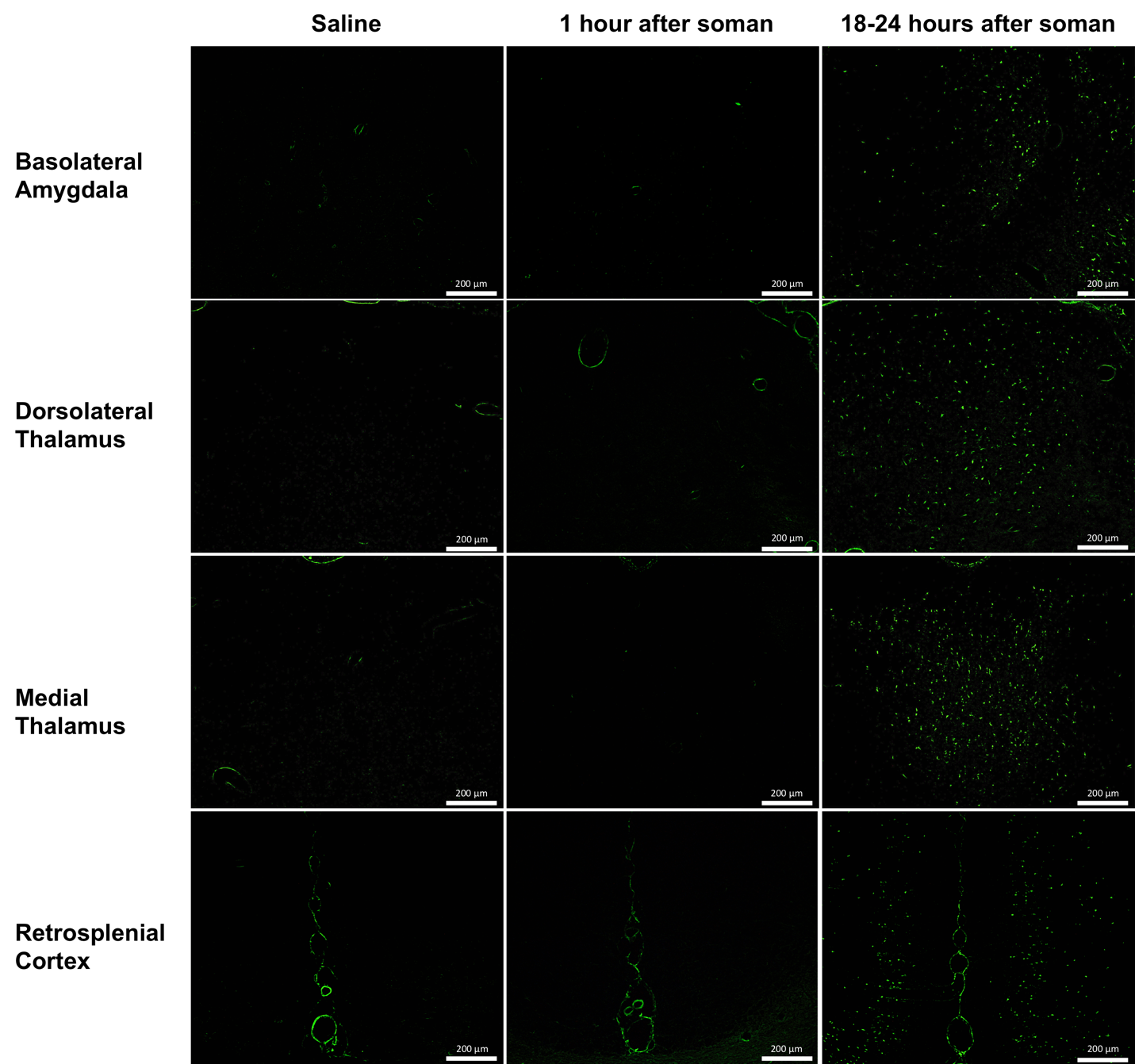


**Supplementary Figure S2.** The effects of soman-induced seizures on neuronal cells following 18-24 hours after soman exposure in the basolateral amygdala, dorsolateral thalamus, medial thalamus, and retrosplenial cortex. There was extensive Fluoro-Jade C staining indicative of neurodegeneration at 18-24 hours after soman exposure.

**
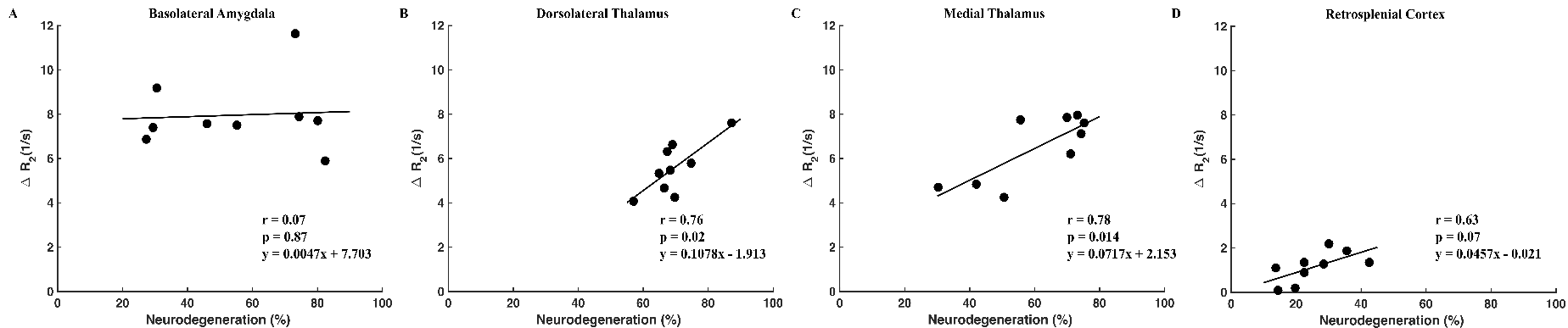
**

**Supplementary Figure S3.** The rate of relaxation (R_2_) was compared to the percentage of neurodegeneration in the basolateral amygdala, dorsolateral thalamus, medial thalamus, and retrosplenial cortex. A-D, there was no significant correlation in the basolateral amygdala (r = 0.07, p = 0.87), and retrosplenial cortex (r = 0.63, p = 0.07). There was a significant correlation in the dorsolateral thalamus (r = 0.76, p = 0.02), and medial thalamus (r = 0.78, p = 0.014). Each point represents a rat.
